# In-Doped ZnO Hexagonal Stepped Nanorods and Nanodisks as Potential Scaffold for Highly-Sensitive Phenyl Hydrazine Chemical Sensors

**DOI:** 10.3390/ma10111337

**Published:** 2017-11-21

**Authors:** Ahmad Umar, Sang Hoon Kim, Rajesh Kumar, Mohammad S. Al-Assiri, A. E. Al-Salami, Ahmed A. Ibrahim, Sotirios Baskoutas

**Affiliations:** 1 Department of Chemistry, Faculty of Science and Arts, Najran University, P.O. Box 1988, Najran 11001, Saudi Arabia; semikim77@gmail.com (S.H.K.); ahmedragal@yahoo.com (A.A.I.); 2 Promising Centre for Sensors and Electronic Devices (PCSED), Najran University, P.O. Box-1988, Najran 11001, Saudi Arabia; msassiri@gmail.com; 3 PG Department of Chemistry, JCDAV College, Dasuya 144205, India; rk.ash2k7@gmail.com; 4 Department of Physics, Faculty of Science and Arts, Najran University, P.O. Box 1988, Najran 11001, Saudi Arabia; 5 Department of Physics, Faculty of Science, King Khalid University, P.O. Box-9004, Abha 61413, Saudi Arabia; Salami11@gmail.com; 6 Department of Materials Science, University of Patras, 26504 Patras, Greece

**Keywords:** In-doped ZnO, stacked nanorods, flower-shaped, sensing, phenyl hydrazine

## Abstract

Herein, we report the growth of In-doped ZnO (IZO) nanomaterials, i.e., stepped hexagonal nanorods and nanodisks by the thermal evaporation process using metallic zinc and indium powders in the presence of oxygen. The as-grown IZO nanomaterials were investigated by several techniques in order to examine their morphological, structural, compositional and optical properties. The detailed investigations confirmed that the grown nanomaterials, i.e., nanorods and nanodisks possess well-crystallinity with wurtzite hexagonal phase and grown in high density. The room-temperature PL spectra exhibited a suppressed UV emissions with strong green emissions for both In-doped ZnO nanomaterials, i.e., nanorods and nanodisks. From an application point of view, the grown IZO nanomaterials were used as a potential scaffold to fabricate sensitive phenyl hydrazine chemical sensors based on the I–V technique. The observed sensitivities of the fabricated sensors based on IZO nanorods and nanodisks were 70.43 μA·mM^−1^·cm^−2^ and 130.18 μA·mM^−1^·cm^−2^, respectively. For both the fabricated sensors, the experimental detection limit was 0.5 μM, while the linear range was 0.5 μM–5.0 mM. The observed results revealed that the simply grown IZO nanomaterials could efficiently be used to fabricate highly sensitive chemical sensors.

## 1. Introduction

Phenyl hydrazine is used for the synthesis of agricultural pesticides and insecticides, in pharmaceutical industries, rocket propellant, and explosives. Even a very low concentration of it may prove very fatal and produces drastic environmental and health hazards. The exposure of phenyl hydrazine may lead to dermatitis, skin irritation, liver and kidney damage, and hemolytic anemia [1]. According to World Health Organization and Environmental Protection Agency, phenyl hydrazine is carcinogenic and has been classified as group B2 human carcinogen [2]. Thus, a fast, reliable, highly sensitive, and selective analytical method is desired to detect even traces of phenyl hydrazine.

Modifications of the structural, morphological, optical, chemical, electronic, and physicochemical properties of the ZnO semiconductor nanomaterials either through doping or through the formation of composites have always attracted the researcher to synthesize such materials [3,4,5,6,7,8,9]. Various doped ZnO nanomaterials have been recently synthesized and explored for their photocatalytic [10,11,12], gas sensing [13,14], photonic crystals [15], spintronics [16], electrochemical sensing [17,18,19], optoelectronics [20,21], dye-sensitized solar cells [22], light emitting diodes [23], field emission transistors [24,25], and many more applications. Methods like sol-gel [24,26], hydrothermal [18,27], ceramics vapor deposition [28], spin coating [29], solution combustion [30], RF sputtering [31], pulse laser deposition [32,33], etc. are reported for the synthesis of doped ZnO nanostructures. However, through the thermal evaporation technique, the directional growth of the nanostructures can be easily controlled by controlling the temperature and source material in contrast with the solution method in which the growth of nanomaterials are depends upon the pH, concentration of the source materials, growth time, template/capping agent/directing agents, etc. Moreover, the use of pure metals as source material grows the nanostructures of high purity and crystallinity.

ZnO is an II–VI, n-type semiconductor with a wide direct band gap of ~3.27 eV, large exciton binding energy (~60 meV), and high electron mobility of 115–155 cm^2^·V^−1^·s^−1^ at room temperature [34,35,36]. Doping with higher valence impurities, such as In^3+^, further enhances the conductivity [37,38]. It has been reported that both the conduction band, as well as the valence band energies of the ZnO, are shifted to lower energy levels while there is no appreciable change in the band gap energy [39]. Additionally, as the ionic radius of In^3+^ (0.094 nm) is greater than that of Zn^2+^ ions (0.074 nm), the incorporation of the In^3+^ ions into the ZnO crystal lattice further produces tensile strains leading to higher surface defects [40]. These properties make In-doped ZnO nanostructures suitable candidates for the fabrication of electrode for the gas, electrochemical sensing, and solar cell applications.

Chava et al. [35] synthesized In-doped ZnO nanoparticles through a cost-effective, low-temperature aqueous solution method, and explored them for the fabrication of photoanode in dye-sensitized solar cells. In-doped ZnO, photoanode exhibited a high short-circuit photocurrent density of 12.58 mA/cm^2^ and a power conversion efficiency of 2.7% as compared to the current density of 8.02 mA/cm^2^ with 1.8% efficiency for the bare ZnO nanoparticles. Badadhe et al. [41] observed a very high gas response and short response and recovery times of 13,000, ~2 s and ~4 min, respectively, for 1000 ppm H_2_S at 250 °C through 3 at. %. In-doped ZnO thin films deposited onto pre-cleaned glass substrates through a conventional spray pyrolysis technique. However, for 50 ppm CO gas, 1 at. % and 2 at. % In-doped ZnO nanostructures prepared by Dhahri et al. [42] showed an excellent gas response with short response and recovery times as compared to pure ZnO at 300 °C. Wang et al. [43] explored the ethanol gas sensing applications of In-doped three-dimensionally ordered macroporous (3DOM) ZnO structures synthesized via a colloidal crystal templating method. High selectivity and sensitivity of the 5 at. % In-doped ZnO structures as compared to pure 3DOM ZnO was attributed to the increased surface area, the increased electron carrier concentration due to the replacement of the Zn^2+^ ions from the crystal lattice by In^3+^ ions, along with the higher rate of O_2_ adsorption. Ge et al. [44] observed significant responses towards trace of various nitro-explosive vapors, including trinitrotoluene (TNT), dinitrotoluene (DNT), para-nitrotoluene (PNT), picric acid (PA), and Research Department Explosive (RDX). At room temperature using 5% In-doped ZnO nanoparticles based gas sensors. The use of metal oxide nanomaterials modified electrodes, through electrochemical sensing analysis, is preferred over other traditional methods due to the excellent reliability, high sensitivity, and short response and recovery times [45].

In this paper, a facile thermal evaporation method is reported for the growth of In-doped ZnO (IZO) with two distinct morphologies as a function of reaction conditions on a silicon substrate. The grown nanomaterials were characterized for their morphological, structural, optical, and crystal properties through relevant characterization techniques. Further, the grown IZO nanomaterials were utilized as efficient electron mediators for the fabrication of phenyl hydrazine chemical sensor. Finally, a comparison was made between the In-doped ZnO nanorods and nanodisks for the electrochemical sensing properties towards phenyl hydrazine.

## 2. Experimental Details

### 2.1. Growth of In-Doped ZnO Nanostructures

The stepped hexagonal nanorods and disk-shaped IZO nanomaterials were grown on silicon substrate by thermal evaporation of metallic zinc (Zn) and indium (In) powders in the presence of oxygen. For the growth of stepped hexagonal nanorods, metallic Zn and In powders were mixed well in the ratio of 5:2, while for the disk-shaped structures, the Zn and In powders were mixed in the ratio of 10:3. Both of the mixtures were separately transferred to the ceramic boats and were placed at the center of the high-temperature ceramic tube furnace. Two different experiments were performed for the growth of stepped hexagonal nanorods and disk-shaped structures. Prior to the experiments, the Si(100) substrates were cleaned with DI water, ethanol, and acetone, sequentially and were dried by nitrogen gas. For both of the experiments, several pieces of Si(100) were placed adjacent to the source boat and ceramic tube furnace chamber was down to 1 torr using a rotary vacuum pump. For the nanorods growth, the reaction was done at 700 °C, while disks were grown at 850 °C under the continuous flow of high purity nitrogen and oxygen gases with the flow-rates of 40 and 100 sccm, respectively. The reaction was terminated in 1.5–2.5 h. After completing the reaction for the desired time, the furnace was allowed to cool to room temperature and the deposited materials were characterized in terms of their morphological, structural, and optical properties.

### 2.2. Characterization Techniques

The necessary characterization techniques were utilized for the evaluation of the morphological, structural, optical, compositional, and crystal phases of the as-synthesized IZO nanostructures. Field emission scanning electron microscopy (FESEM; JEOL-JSM-7600F, JEOL, Tokyo, Japan) attached with energy dispersive spectroscopy (EDS) explored the morphological, structural, and compositional characteristics. In-doped ZnO nanostructures were subjected to X-ray diffraction (XRD; JDX-8030W, JEOL, Tokyo, Japan) analysis using Cu-Kα radiation (λ = 1.54 Å) in the diffraction angle range of 20–65° elaborated the crystal structure, purity, crystal sizes and crystallinity. The optical properties of the deposited materials were examined by room-temperature photoluminescence (PL), measured with the He-Cd (325 nm) laser lines as the exciton source.

### 2.3. Fabrication of Phenyl Hydrazine Electrochemical Sensor Based on IZO Nanomaterials

To fabricate the phenyl hydrazine chemical sensors, firstly, the grown IZO nanomaterials were transferred from the substrates to the glassy carbon electrodes (GCE) and wetted by phosphate buffer solution (0.1 M PBS) with pH = 7.4 and dried gently with the flow of high purity nitrogen gas.

The PBS (0.1 M, pH = 7.4) solution was prepared by mixing 0.2 M disodium phosphate (Na_2_HPO_4_) and 0.2 M monosodium phosphate (NaH_2_PO_4_) solution in 100 mL de-ionized water. Consequently, 5 weight % Nafion solution was dropped onto the modified electrode to form a net-like structure on the electrode, which can hold the functional materials during the sensing measurements. Prior to the experiments, the GCE was polished with alumina slurry and was then sonicated in de-ionized water. The modified electrode, i.e., nafion/In-doped ZnO/GCE was finally dried at 40 °C for 12 h. All of the electrochemical experiments were performed at room temperature by Keithley 6517A-USA (Tektronix, OR, USA) electrometer with computer interfacing with a simple current-voltage (I–V) technique. For the two electrode system, the modified electrodes were used as working electrode, in which the Pt wire was employed as a counter electrode. The sensitivity of the fabricated sensors was determined by plotting a calibration curve of current vs. concentration.

## 3. Results and Discussion

### 3.1. Characterization of IZO Nanostructures

**Figure 1:**
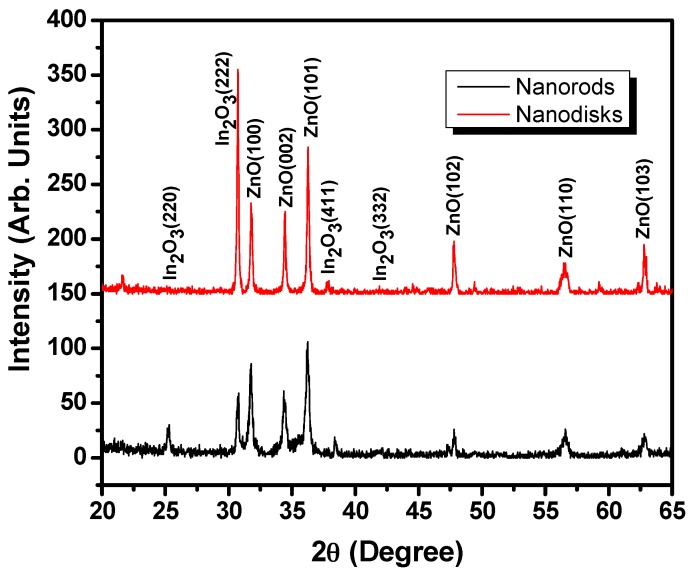
X-ray diffraction (XRD) diffraction patterns for In-doped ZnO (IZO) nanorods and nanodisks.

The crystallinity and crystal properties of as-grown IZO nanomaterials were examined by X-ray diffraction and the observed results are shown in Figure 1. It was observed that the stepped hexagonal IZO nanorods exhibited several sharp and well-defined diffractions peaks corresponding to the diffraction planes of (100), (002), (101), (102), (110), and (103) at diffraction angles of 31.79°, 34.35°, 36.23°, 47.79°, 56.57°, and 62.81°, respectively. However, the IZO nanodisks show several well-defined diffraction peaks appeared at 2θ = 31.77°, 34.45°, 36.27°, 47.79°, 56.43°, and 62.79°, corresponding to the diffraction planes of (100), (002), (101), (102), (110), and (103), respectively. All of the observed diffraction peaks are well matched with the characteristic peaks of wurtzite hexagonal phase of ZnO. The observed diffraction peaks are well-matched with the JCPDS data card no. 36-1451 and the reported literature [18,45,46,47]. The incorporation of the In^3+^ ions into the crystal lattice of the ZnO can be confirmed by the presence of additional peaks for In_2_O_3_ corresponding to diffraction planes (220), (222), (411), and (332) [48,49,50]. However, the peak for diffraction planes (220) was of very low intensity in case of IZO nanorods, which may be due to the very low concentration of the In^3+^ ions.

The crystallite sizes (d) of the thermally deposited IZO nanostructures were evaluated by using Debye–Scherrer equation (Equation (1)) [18,47].

(1) $$d=\frac{0.89\lambda }{\beta .\mathrm{Cos}\theta }$$

where, λ = the wavelength of X-rays used (Cu-Kα radiation with λ = 1.54 Å), θ is the Bragg diffraction angle and β is the peak width at half maximum (FWHM). Three most intense peaks were considered for the calculation of FWHM, and thus the crystallite sizes of the IZO nanorods and nanodisks and the corresponding results are represented in Table 1. The average crystallite sizes of 24.80 nm and 42.24 nm were calculated for the In-doped ZnO nanorods and nanodisks, respectively.

**Figure 2:**
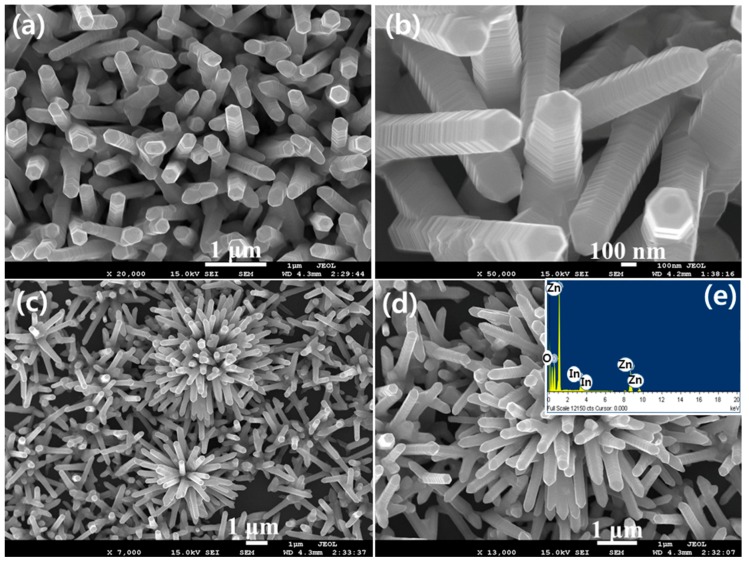
(**a**,**b**) High magnification (**c**,**d**) low magnification Field emission scanning electron microscopy (FESEM) images and (**e**) EDS spectrum of stepped hexagonal IZO nanorods synthesized at the 700 °C growth temperature.

The morphology of the IZO nanostructures thermally synthesized at 700 °C is shown in Figure 2a–d. Stepped hexagonal nanorod shaped morphologies with uniform shape and sizes but with orientations in different directions can be clearly seen from low (Figure 2c,d) as well high (Figure 2a,b) magnification FESEM images. Some of these nanorods seem to be originated from a common base resulting in an urchin shaped structures. The average length of the nanorods was ~1.5 μm, however, the diameter was not uniform. Each IZO nanorod further showed a hexagonal cross-section with a thickness of ~150 nm (Figure 2b). The elemental composition of the IZO nanorods, as examined by the EDS attached with FESEM is shown in Figure 2e. The IZO nanorods are made of Indium, Zinc, and oxygen only. No other peak corresponding to any elemental impurity, prove that thermally synthesized nanorods are of high purity.

**Figure 3:**
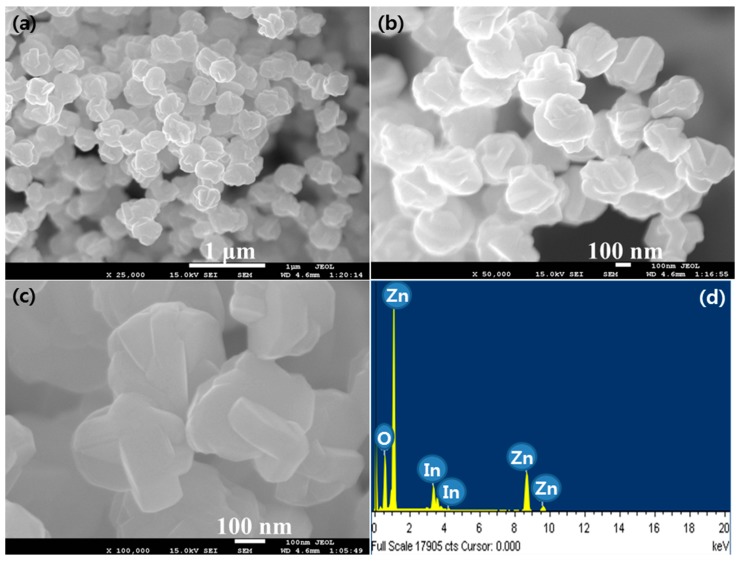
(**a**,**b**) Low magnification (**c**) high magnification FESEM images and (**d**) energy dispersive spectroscopy (EDS) spectrum of IZO nanodisks synthesized at the 850 °C growth temperature.

The morphology of the IZO nanostructures thermally synthesized at 850 °C is shown in Figure 3a–c. Low magnification FESEM images shown in Figure 3a,b exhibit disk shaped morphologies. However, high magnification FESEM images, as shown in Figure 3c, revealed that the disk-shaped morphologies are further composed of hook-shaped structures that are interlocked with each other. The average diameter of disk shaped morphology was ~250–300 nm. Figure 3d shows the EDS spectrum for the IZO nanodisks, which confirmed the purity of thermally deposited nanodisks as peaks for Indium, Zinc, and oxygen atoms are present.

**Figure 4:**
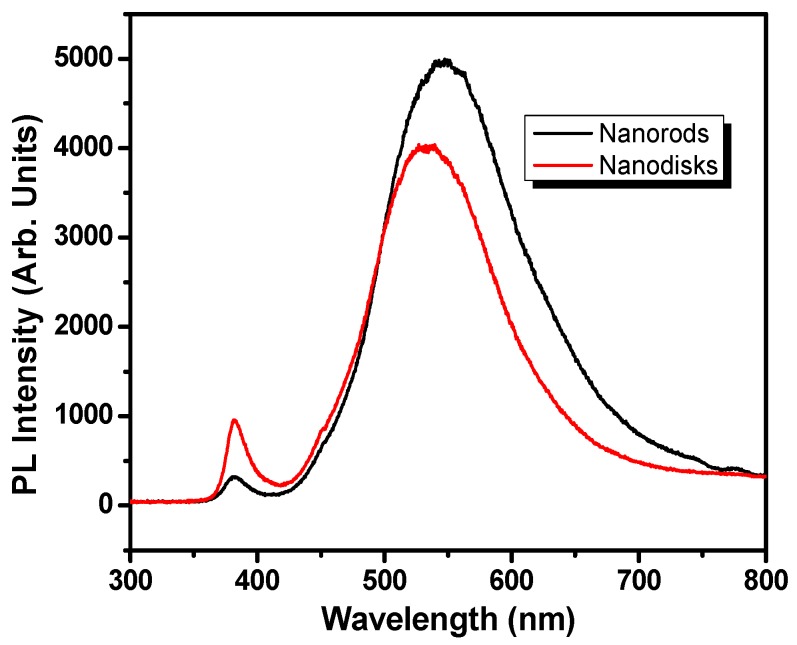
Typical room-temperature photoluminescence (PL) spectra of stepped hexagonal nanorods and flower-shaped IZO nanomaterials.

In order to evaluate the structural and optical property of the thermally deposited In-doped ZnO nanostructures, Photoluminescence spectroscopic analysis was performed at room temperature using a He-Cd source having 325 nm excitation wavelength. The corresponding PL spectra are shown in Figure 4. Strong UV emission peaks centered at 381.6 (3.249 eV) and 381.4 nm (3.251 eV) were observed for In-doped ZnO nanorods and nanodisks, respectively. These peaks may be attributed to the near band edge emission (NBE) resulting due to recombination of free excitons as well as to the transition from 1 longitudinal optical (LO)-phonon replica of two electron satellites (TES) lines of ZnO [51,52,53,54]. A slight shift in band edge peak with a significant increase in the UV emission intensity for IZO nanodisks as compared to nanorods may be attributed to the better crystallinity of former, which is also confirmed by the XRD analysis (Figure 1). Additionally, broad but strong bands in the visible region with emission peaks at 547.5 nm (2.264 eV) and 539.4 nm (2.299 eV) for IZO nanorods and nanodisks, respectively, were also observed and can be assigned to is due to the superposition of green, yellow-orange, and red emissions [51,55,56]. The green emission bands for both the morphologies originate due to the radial recombination of a photogenerated positively charged hole (h^+^) with a negatively charged electron (e^−^) of the singly ionized oxygen (O) vacancies on ZnO surface lattice [57,58].

### 3.2. Electrochemical Sensing Applications of IZO Nanostructures

IZO nanostructures based electrochemical sensors were tested for the detection 0.05 μM phenyl hydrazine in 0.1 M phosphate buffer solution (PBS) with pH = 7, as compared to blank PBS.

**Figure 5:**
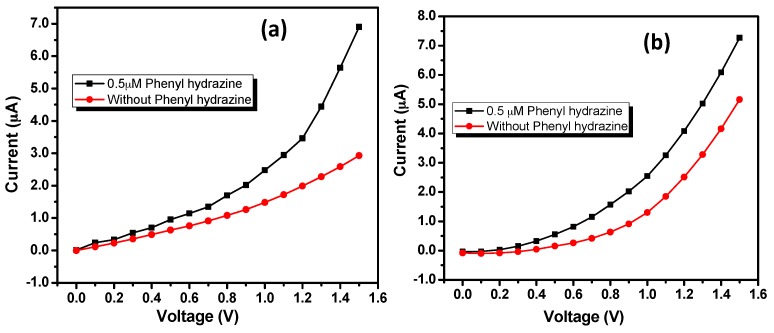
I–V response with 0.5 µM phenyl hydrazine and without phenyl hydrazine using IZO (**a**) nanorods and (**b**) nanodisks modified GCE in 0.1 M PBS solution.

The current responses were measured from 0.0 to +1.5 V. Significant increase in the current response for even very low concentration of 0.05 μM phenyl hydrazine as compared to blank PBS confirms that both the doped ZnO nanostructures could act as efficient electron mediators and electro-catalysts for the electrochemical detection of phenyl hydrazine at room temperature (Figure 5a,b). For both of the sensors, a continuous increase in the current response was observed with an increase in the potential applied. At +1.5 V current responses of 7.268 μM and 6.903 μM were recorded, respectively, for IZO nanorods and nanodisks based sensors.

**Figure 6:**
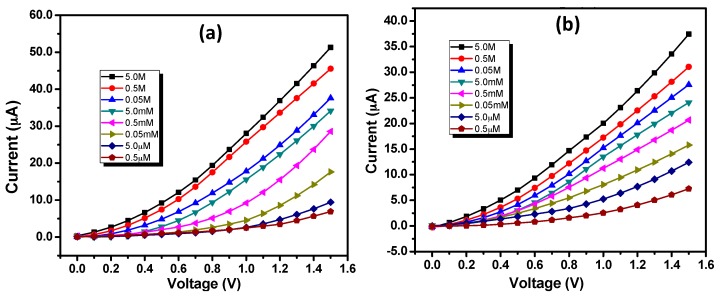
I–V responses for IZO (**a**) nanorods and (**b**) nanodisks against various concentrations of phenyl hydrazine (0.5 μM–5.0 M) in 0.1 M phosphate buffer solution (PBS).

For the estimation of important sensing parameters like sensitivity, linear dynamic range, detection limit, and correlation coefficient, a series electrochemical sensing experiments were conducted using both the IZO nanostructured modified electrodes using different concentrations. A series of phenyl hydrazine solutions with a concentration range of 0.5 µM–5.0 M in 0.1 M PBS were prepared. Figure 6a,b represent the I–V response curves for IZO nanorods and nanodisks modified GCE, respectively, against various concentrations of phenyl hydrazine in 0.1 M PBS. Expectedly, a continuous increase in current response is seen with the sequential increase in the phenyl hydrazine concentrations from 0.5 μM to 5.0 M in 0.1 M PBS. This may be attributed to the generation of a large number of ions due to ionization of phenyl hydrazine into resulting in large ionic strength at higher concentrations [45,59]. Current responses of 33.56 μA and 51.29 μA were observed for 5.0 M concentrations of phenyl hydrazine in 0.1 M PBS at +1.5 V using GCE modified with IZO nanorods and nanodisks, respectively. Thus, from these results, it can be concluded that IZO nanodisks modified GCE exhibits better sensing performances than stepped hexagonal IZO nanorods modified GCE.

**Figure 7:**
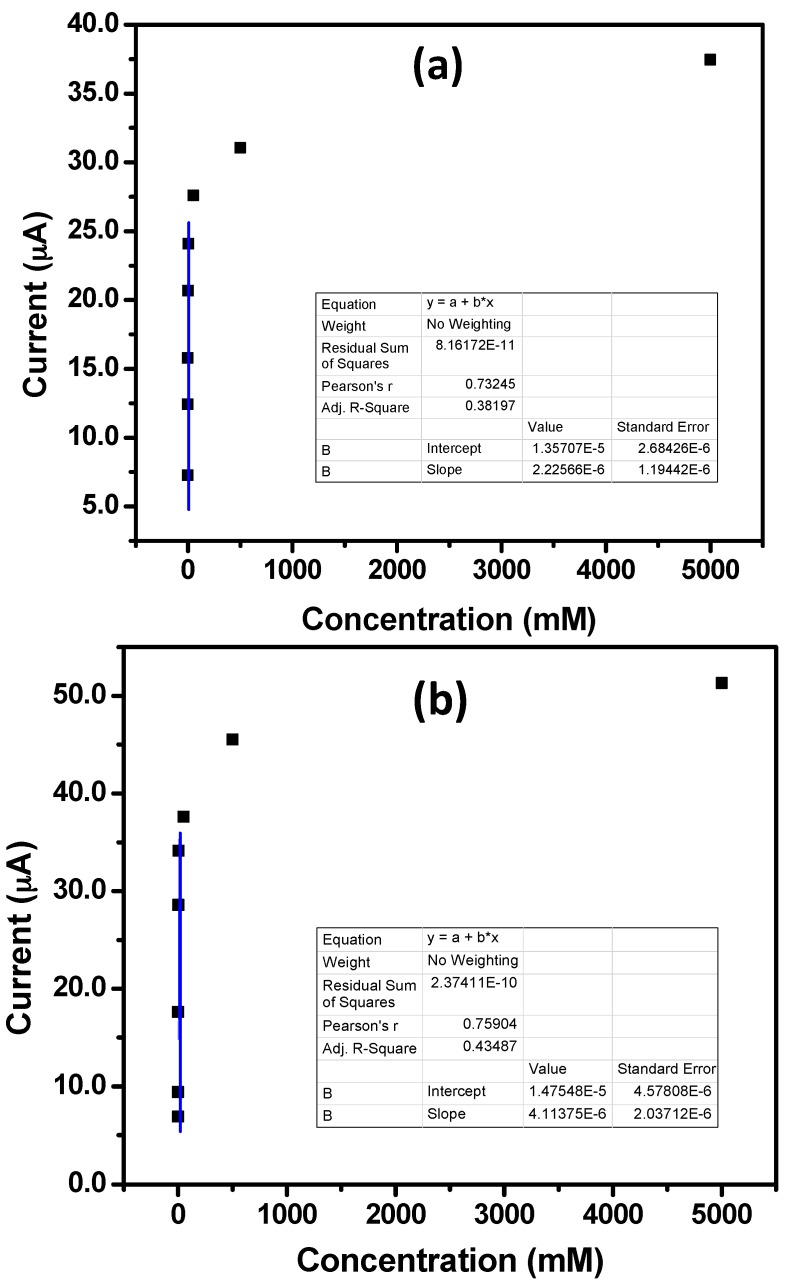
Calibration curves for IZO (**a**) nanorods and (**b**) nanodisks against various concentrations of phenyl hydrazine (0.5 μM–5.0 M) in 0.1 M PBS.

Figure 7 represents the respective calibration plots for IZO nanostructures. Sensitivity, linear dynamic range (LDR) and detection limits were evaluated from these calibration plots. Sensitivity was measured from the ratio of the slope of the calibration plot to the active surface area of the modified GCE [18,45]. For stepped hexagonal IZO nanorods modified GCE, sensitivity was 70.43 μA·mM^−1^·cm^−2^, whereas for IZO nanodisks modified GCE it was 130.18 μA·mM^−1^·cm^−2^. For both of the modified electrodes the LDR and experimental detection limits were 0.5 μM–5.0 mM and 0.5 μM, respectively. The high sensitivity of IZO nanodisks based sensors are due to the high surface to volume ratio of as-grown nanodisks when compared to the IZO nanorods. The low-dimensionality and reduced dimensions of the IZO nanodisks can be well-understood by the observed SEM images, as shown in Figure 2 and Figure 3. Thus, it can be concluded that nanomaterials dimensions are significantly important for the sensing performance, and hence with reduced dimensions, enhanced sensitivity for the fabricated sensor based on IZO nanodisks can be achieved.

It has been reported that the greater the growth temperature the greater the density of defects on the surface of the nanomaterials due to the diffusion of the oxygen from the crystal lattices creating anion vacancies and enhancing the positive charge density [60,61,62]. As IZO nanodisks were grown at higher temperature i.e., 850 °C as compared to a 700 °C growth temperature for In-doped ZnO nanorods, the former exhibited superior sensing behavior.

The phenyl hydrazine responses for different sensors reported in the literature are summarized in Table 2. As grown IZO nanodisks based phenyl hydrazine sensor exhibited better sensitivities as compared to other reported sensors. Thus, IZO nanodisks could be efficient electron mediator and electro-catalyst for the fabrication of phenyl hydrazine chemical sensors.

### 3.3. Proposed Mechanism

Doping of the ZnO nanostructures with In^3+^ ions induces the surface lattice defects along with an active surface area for the effective adsorption of the O_2_ molecules from the surrounding air, as well as the phenyl hydrazine molecules from the PBS [71,72]. The presence of electron donor –NH_2_ groups and π-electron density of the phenyl rings of phenyl hydrazine molecules further enhance the attractions between the analyte molecules and the active sites of the IZO nanostructures. The adsorbed O_2_ molecules are subsequently converted into oxygenated anionic species i.e., O_2_^−^, O^2−^, and O^−^ etc. by extracting the conduction band electrons of the IZO nanostructures [73] (Equations (2)–(4)).

(2) $$O_{2\;\left(g\right)}+{\;e}^{-}↔O_{2\;\left(\mathrm{ads}\right)}^{-}$$

(3) $$O_{2\;\left(g\right)}+\;2e^{-}↔2O_{\left(\mathrm{ads}\right)}^{-}$$

(4) $$O_{2\;\left(g\right)}+\;4e^{-}↔2O_{\left(\mathrm{ads}\right)}^{2-}$$

**Figure 8:**
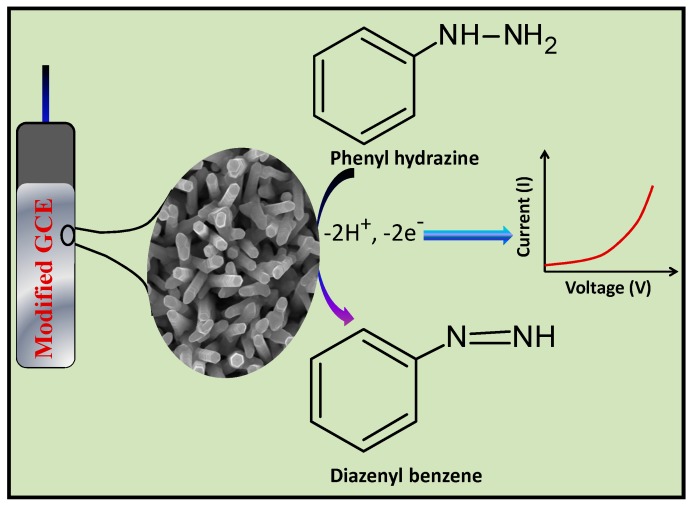
Proposed mechanism for phenyl hydrazine chemical sensing based on IZO nanostructures coated GCE by I–V technique.

These oxygenated chemical species oxidize the phenyl hydrazine molecules into diazenyl benzene (Figure 8). This oxidation process releases the electrons that are transferred back to the conduction band of the IZO nanostructures, thereby increasing the conductivity and hence the response current.

## 4. Conclusions

In summary, In-doped ZnO (IZO) nanomaterials, i.e., stepped hexagonal nanorods and nanodisks were grown on silicon substrate by simple thermal evaporation process and characterized in detail using several techniques. The detailed morphological studies confirmed that both IZO nanomaterials possess well-crystallinity with wurtzite hexagonal phase and grown in high density. A suppressed UV emissions and strong green emissions for both IZO nanomaterials, i.e., nanorods and nanodisks were seen in the room-temperature PL spectra. The fabricated phenyl hydrazine chemical sensors based on as-grown IZO nanomaterials exhibited high sensitivities, i.e., 70.43 μA·mM^−1^·cm^−2^ and 130.18 μA·mM^−1^·cm^−2^, respectively, for nanorods and nanodisks. The experimental detection limits for both of the sensors were 0.5 μM, while the linear ranges were 0.5 μM–5.0 mM.

## Figures and Tables

**Table 1:** The peak width at half maximum (FWHM) values and crystallite sizes of IZO nanostructures.

S.N.	(hkl)	IZO Nanorods			IZO Nanodisks		
		2θ (°)	FWHM (β)	Crystallite Size (nm)	2θ (°)	FWHM (β)	Crystallite Size (nm)
1.	(100)	31.79	0.29564	27.65	31.77	0.19751	41.39
2.	(002)	34.35	0.35795	22.99	34.45	0.1810	45.48
3.	(101)	36.23	0.34833	23.75	36.27	0.20768	39.84

**Table 2:** Reported phenyl hydrazine sensing parameters of various nanostructures.

Sensing Materials	Sensitivity (μA·mM^−1^·cm^−2^)	LDR (μM–mM)	Detection limit (μM)	Ref.
ZnO nanourchin	42.1	98.0–3.126	78.6	[45]
ZnO–SiO_2_ nanocomposite	10.80	390.0–50.0	1.42	[63]
ZnO-Fe_2_O_3_ microwires	8.33	10^−3^–10.0	6.7 × 10^−4^	[64]
Al- doped ZnO Nanoparticles	1.143	10.0–50.0	1.215 ± 0.02	[65]
CuO hollow spheres	0.578	5 × 10^3^–10.0	2.4 × 10^3^	[66]
CuO flowers	7.145			
Fe_2_O_3_ nanoparticles	57.88	97.0–1.56	97	[67]
Cd_0.5_Mg_0.5_Fe_2_O_4_ ferrite nanoparticles	7.01	3 × 10^3^–100	3 × 10^3^	[68]
TiO_2_ nanotubes	40.9	0.25–0.10	0.22	[69]
Ferrocene-modified carbon nanotube	25.3	0.85–0.7	0.6	[70]
*IZO nanorods*	*70.43*	0.5–5.0	0.5	*This study*
*IZO nanodisks*	*130.18*			
